# Correlation Analysis between Central Corneal Thickness and Intraocular Pressure in Juveniles in Northern China: The Jinan City Eye Study

**DOI:** 10.1371/journal.pone.0104842

**Published:** 2014-08-22

**Authors:** Wen Wei, Zhaoshan Fan, Lihua Wang, Zhiwei Li, Wanzhen Jiao, Yun Li

**Affiliations:** Shandong Provincial Hospital Affiliated to Shandong University, Jinan, China; Bascom Palmer Eye Institute, University of Miami School of Medicine, United States of America

## Abstract

**Purpose:**

To determine the distributions and relation of central corneal thickness (CCT) and intraocular pressure (IOP) by NT-530P in Chinese juveniles, and the effect of gender, age, height, weight and refractive errors on the CTT and IOP.

**Methods:**

CCT and IOP of 982 eyes in 514 juveniles aged from 7 to 18 years were measured with NT-530P. Multi-linear regression and ANOVA analysis were used to analyze the relation of CCT and IOP, and the effect of gender, age, height, weight, refractive condition on CCT and IOP respectively.

**Results:**

The mean CCT and IOP were 554.19±35.46 µm and 15.31±2.57 mmHg. There were significant correlations between the CCT and IOP values. Linear regression analysis revealed a positive correlation between CCT and IOP (r = 0.44, P<0.05). Linear regression equation: IOP = −2.35+0.032CCT, which means the IOP will increase 0.32 mm Hg for every 10-µm increase in CCT. The mean of Corrected IOP (CIOP) was 15.32±2.38 mmHg and had no relation with CCT. There was a negative correlation between refraction degree and CCT (P<0.05), but no correlation between refraction degree and IOP. Multi-linear regression model revealed that the height, weight, age and gender have no effect on the distribution of CCT and IOP respectively.

**Conclusions:**

There is a 0.32 mmHg increase in IOP for every 10-µm increase in CCT. The height, weight, age and gender has no effect on the distribution of CCT and IOP. CCT will become thinner with myopia diopters increases in juveniles. The measurement of CCT is helpful in evaluating the actual IOP correctly.

## Introduction

With the rapid development of refractive surgery, central corneal thickness (CCT) has become an important parameter for choosing surgery modality and assessing prognosis. Meanwhile CCT in the diagnosis of glaucoma field also has an important role [1]. Previous studies have revealed the positive relationship between CCT and IOP among adults. Every 10 µm increase in CCT leads to 0.15–1.0 mmHg increase in IOP [2], [3], [4]. The CCT as well as IOP is important for assessing the glaucoma considering the low CCT will lead to the underestimation of IOP and interfere the prognosis of glaucoma [5]. To date, scare reference was found to elaborate the relation of CCT and IOP in Chinese school children. This study aims to evaluate the relation of CCT and IOP in Chinese school children aged from 7 to 18 years, and elaborate the effect of gender, age, height, weight and refractive errors on the relation of CCT and IOP. In this study, CCT and IOP were measured using the Tonopachy NT-530P (Nidek, Gamagori, Japan). Tonopachy NT-530P combines a non-contact tonometer and pachymeter into one unit, by which providing the advantage of two types of measurements at one time [6]. NT-530P automatically measures CCT in each subject using the principle of the Scheimpflug camera system following the same principle as the Pentacam [6], [7] and measures IOP like a conventional non-contact tonometer which uses a puff of air to flatten the cornea. It provides IOP and CIOP which is corrected according to CCT at the same time.NT-530P offers a non-invasive CCT and IOP measurement in a single unit and reduces a patient's discomfort with continuously measurements. This advantage is more suitable for children than Goldman.

## Materials and Methods

### Ethics Statement

This was a prospective cross-sectional study conducted as part of an eye health screening project among school children from Shandong Normal University Affiliated Primary School, Jinan No. 11 middle school, Jinan Dianliu high school in Shandong province and Shandong Medical College, which locate in Jinan city of Shandong province of northern China. The study was conducted in the Department of Ophthalmology, Shandong Provincial Hospital Affiliated to Shandong University. Written informed consent was obtained from the parents of all children prior to the initiation of the study. The Declaration of Helsinki was adhered to in all procedures and the approval of the ethics committee of Shandong Provincial Hospital Affiliated to Shandong University was obtained before the initiation of study.

### Subjects

The study was performed from January10 to February 5, 2013. Children aged from 7 to 18 years old without systemic and ocular disease except refractive error were recruited in this study. Children with IOP greater than 21 mmHg, glaucoma and its family history, corneal disease, intraocular surgery, cataract, eyelid abnormality, history of prematurely, were excluded [8]. Children likely to have abnormally thin corneas such as those with Marfan or any other systemic abnormality, were also excluded. The corrected visual acuity of participants is more than or equal to 20/20.

### Methods

All involved cases underwent the examination of slit lamp and fundoscopy, and the assessment of visual acuity with a Snellen chart. In the cases of refractive error cycloplegic was used with 3 drops of 1% cyclopentolate (Cyclogyl, Alcon, USA) that were administered 5 minutes apart. After another 40 minutes subjective refraction was measured using an auto refractometer (Topcon KR8100, Topcon, Japan). CCT and IOP were measured using NT-530p (Nidek, Japan). Height and weight measurements were made using a height and weight scale (Jiangsu Su Hong Company, China).

### Data Handling and Statistical Analysis

SPSS (version 17.0 for Windows) was used for data analysis. The Kolmogorov-Smirnov test was used for distribution evaluation. To compare CCT and IOP values between the age, gender, height, weight and equivalent spherical degree of juveniles, for each parameter (i.e. CCT, IOP, or CIOP), t test, Wilcoxon rank test, Kruskal-Wallis test, and one-way ANOVA variance analysis was used based on the homogeneity of variances of the data set. Linear regression analysis was used to evaluate the relationship between CCT and IOP. Multivariate linear regression was performed to explore the association between the IOP (dependent factor) and age, gender, height, weight and equivalent spherical degree (independent factor) on right and left eyes, respectively. Data are presented as means ± standard deviation, *P*<0.05 was considered statistically significant.

## Results

### General information

**Figure 1:**
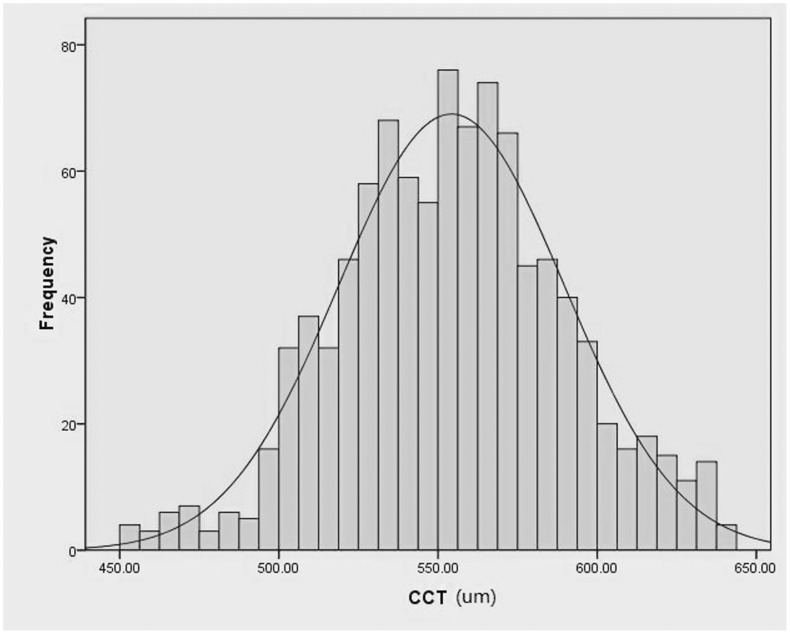
Distribution of central corneal thickness (CCT) in 982 eyes of children aged 7 to 18 years. CCT is normally distributed. The mean CCT was 554.19±35.46 µm.

In total, the study included 982 eyes of 514 healthy school children, in which 446 eyes of 235 children were boys (45.7%) and 536 eyes of 279 children were girls (54.3%). The mean of age, height and weight were 12.94±2.92 years (7–18 years), 1.56±0.08 m (1.19∼1.95 m) and 45.76±12.23 kg (25∼100 kg) respectively. The mean CCT, IOP and CIOP were 554.19±35.46 µm (451–639 um), 15.31±2.57 mmHg (9.5–21.0 mmHg) and 15.32±2.38 mmHg respectively. More details were showed in Table 1. Based on the result of normality test, the CCT followed normal distribution with a mean value of 554.19 µm (Figure 1).

**Table 1:** Mean value of height, weight, CCT, IOL and CIOP in different groups classified with age.

Age (years)	Number (eyes)	Height (m)	Weight (Kg)	CCT (µm)*	IOP (mmHg)*	CIOP (mmHg)*
7∼	153	1.28±0.08	30.06±12.48	560.84±35.34	15.65±2.54	15.11±2.44
10∼	282	1.44±0.08	40.31±12.82	554.43±34.46	15.41±2.48	15.38±2.36
13∼	337	1.65±0.08	50.80±13.38	553.96±32.11	15.72±2.60	15.73±2.41
16∼18	210	1.75±0.09	55.01±13.37	543.53±34.16	15.04±2.44	15.02±2.24

*There was no significant difference among CCT, IOP and CIOP in groups with different age and in groups with different weight and height.

### CCT, IOP and CIOP values, the relationships between these values and gender, age, height and weight

In male and female, CCT were 556.82±35.23 µm and 552.00±35.56 µm, IOP were15.44±2.58 mmHg and 15.25±2.57 mmHg, CIOP were 15.29±2.31 mmHg and 15.35±2.44 mmHg. There was no difference between boys and girls (P>0.05). Subjects were grouped based on different age (7 to 9, 10 to 12, 13 to 15, and 16 to 18 years). There was no significant difference among CCT, IOP and CIOP in groups with different age and in groups with different weight and height(P>0.05) (Table 1). Multiple factors regression was performed on right and left eyes individually using IOP was independent factor and CCT (×1), gender (×2), age (×3), height (×4), weight (×5), equivalent spherical degree (×6) as independent factors IOP has positive relation with CCT (*P*<0.01), and has no relation with gender, age, height, weight and equivalent spherical degree(P>0.05)(Table 2).

**Table 2:** Multiple regression model about IOP and CCT, age, gender, height, weight and Diopter.

IOP		C	CCT (×1)	Gender (×2)	Age (×3)	Height (×4)	Weight (×5)	Diopter (×6)
Left Eye	β	−3.561	0.033	0.308	−0.118	0.583	−0.006	−0.006
	P	0.00	0.00	0.52	0.30	0.30	0.46	0.52
Right Eye	β	1.135	0.031	0.539	−0.030	0.853	−0.004	−0.004
	P	0.00	0.00	0.22	0.34	0.56	0.64	0.96

R^2^(left) = 0.18 F(left) = 17.00 R^2^(right) = 0.22 F = 23.779.

### The relationships between CCT and IOP, CIOP values

**Figure 2:**
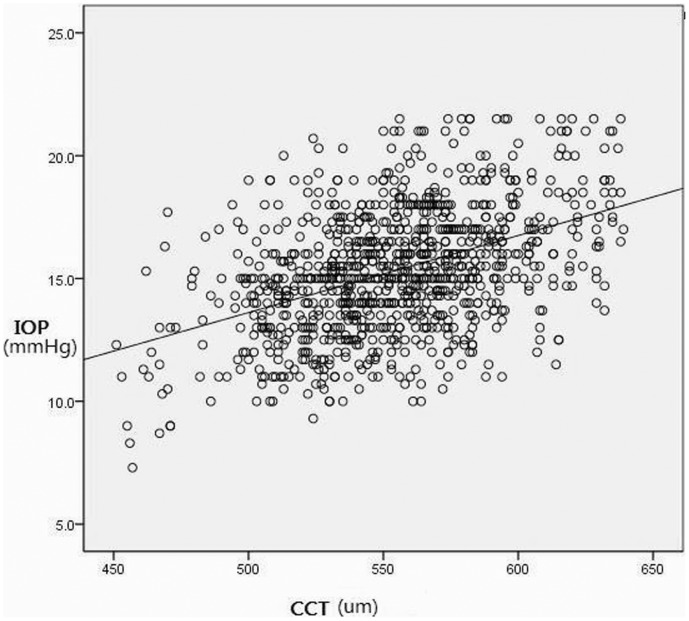
Scattergram of central corneal thickness (CCT) versus intraocular pressure (IOP) of children aged 7 to 18years (r = 0.44, n = 982).

Linear regression analysis revealed a positive relation between the CCT and IOP values (*P*<0.05, r = 0.44) with equation: IOP = −2.35+0.032CCT (Figure 2), which means the IOP will increase 0.32 mm Hg for every 10-µm increase in CCT. There was no significant collection between the CCT and CIOP values.

### The collection between different CCT and IOP, CIOP values

According to Doyle's [9] criteria, CCT values were stratified into three groups: thin cornea group (CCT<520 µm), normal thickness cornea group (CCT = 520–580 µm) and thick cornea group (CCT>580 µm). The IOP values were significant difference among different CCT groups (P<0.05). CCT become thicker with IOP increases. The CIOP values were significant difference among different CCT groups (P<0.05). CCT will become thinner with CIOP increases (Table 3).

**Table 3:** The relationship between different CCT and IOP.

groups	Number (eyes)	CCT (µm)	IOP (mmHg)	CIOP (mmHg)
Thin cornea	156	<520	13.71±2.37	16.37±2.16
Normal cornea	607	520∼580	15.23±2.35	15.40±2.29
Thick cornea	219	>580	16.33±2.57	14.36±2.44

### The relationships between refractive errors and CCT, IOP and CIOP values

The study included 710 eyes of 355 ametropia healthy school children. The mean refractive errors of all subjects was −3.85±2.13D (−0.5D∼−8.75D). According to spherical equivalent (SE) subjects were classified into four groups: emmetropia (+0.5∼−0.5), low myopia (−0.5D≤SE<−3.00 D), moderate myopia (−3.00D≤SE≤−6.00D) and high myopia (>−6.00D). Table 4 shows the results of further analysis yielded no significant findings regarding IOP and CIOP distribution among the different myopia groups (*P*>0.05). There was a negative correlation between CCT and refractive error (P<0.05). CCT decreased with the increasing of myopia diopter.

**Table 4:** The relationships among refractive errors and CCT, IOP and CIOP values.

Dioptre (D)	Number (eyes)	CCT (µm)	IOP (mmHg)	CIOP (mmHg)
+0.50∼−0.50	272	557.98±33.77	15.61±2.57	15.41±2.49
≥−0.50∼−3.00	305	560.55±35.55	15.54±2.53	15.24±2.32
≥−3.00∼−6.00	296	550.75±34.74	15.23±2.49	15.37±2.34
>−6.00∼−9.00	109	547.65±38.09	15.44±2.72	15.99±2.48

## Discussion

### Normal central corneal thickness (CCT) values

Ultrasound pachymetry is the gold standard for measuring CCT. CCT was observed from 523 to 579 µm in different areas of children which supports the hypothesis of the existence of structural variations among different ethnic and racial groups [6], [7], [8], [9], [10], [11]. However there are few studies evaluating CCT with the theory of Scheimpflug camera, especially in children population. The mean CCT revealed in this study was 554.19±35.46 µm in normal population aged 7–18 years, which is consistent with the previous report [12] that mean CCT in southern China children aged 8–16 years was 550.7 µm measured with Pentacam.

The relationship of CCT and age in child population is controversial. Muir et al. [6] reported CCT of children increases until 5 years old, then remains stable, which is followed by a slight decrease from the age of 10 to 14 years old. Hussein [13] suggested that CCT increases slowly with time and reaches adult levels until 5 and 9 years old. While Bradfield [14] reported CCT increases from 1 to 11 years old, while the rate of increase steadily decreases, with year-to-year differences steadily decreasing and reaching a plateau after age 11. Sakalar [15] found CCT reaches adult values around 14 years old. However, others CCT measurements showed no age-related change in healthy children of Czech, Chinese and some other countries [10], [12], [16], [17]. Our results suggested that there is no significant relationship between CCT and age.

Some scholars suggested there is no significant relationship between CCT and gender [10], [17], while other reports regarded CCT is thicker in boys than that of girls [12], [15], [18], [19]. Tong [18] reported CCT is thicker about 6.4 µm in boys than girls. Our results showed the CCT of boys is 4.8 µm thicker than that of girls, however there is no statistical difference. No correlation of height, weight and CCT is found in this study.

The relationship of CCT and refractive errors is controversial. Li Jinghai [20] reported there is a negative correlation between CCT and refractive error. Chang [21] suggested there is relationship between CCT and the type of refractive errors. Axial myopia refractive corneal thickness decreased according to the increasing of refractive error. However Zhang Shisheng [22] suggested there is a positive correlation between CCT and refractive error. Lin et al. [23] suggested there is no significant relationship between CCT and refractive error. Bradfield [14] reported for every degree of increased myopic refractive error, CCT is 1 µm thinner on average. Our results found that there is a negative correlation between CCT and myopia refraction degree.

### Normal intraocular pressure (IOP) values

Normal IOP in adults is 10–21 mmHg. Mean IOP of youth aged from birth to 17 years old measured in different methods was12.0–19.3 mmHg in Europe, Turkey, India, Malaysia, China and other areas [6], [10], [11], [15], [18], [24], [25], [26]. The IOP using NCT in white subjects aged 5–15 years old is around 16 mmHg [27], [28], and similar IOP was observed in Chinese population [26]. The mean IOP values 15.31±2.57 mmHg in our study is consistent with previous reports.

Some reports showed that IOP was significantly higher in girls than in boys [15], while other studies suggested gender were not associated with IOP [16]. The relationship of children between IOP and age is controversial. Some suggested that IOP has positive relation with age [16], [25], [29], while other reports showed age has no influence on IOP [10], [24]. Our study suggested there is no differences between gender, age and IOP, and no differences between weight, height and IOP.

### The relationships between refractive errors and IOP values

The relationship between IOP and diopter is not consistent [30] ^.^ A population-based study in Wisconsin reported that myopia patients were 60% more likely to have glaucoma than emmetropic persons [31]. Similarly, the incidence of myopia in open angle glaucoma, low-tension glaucoma and ocular hypertension is also high [32]. Moderate and especially high myopia is considered a risk factor for the development and the progression of glaucoma [33]. Considering the close relation of IOP and refraction condition,clinicians should attach great importance to the IOP situation of adolescents with ametropia [34].

Previous studies have revealed that IOP and myopia degree has positive relation [31], [35], [36]. The racial background effect the relationship of myopia level and IOP [37]. Our study recruited a total of 514 adolescents aged 7–18 years old, including 710 eyes of 355 myopia patients. No correlation between diopter and IOP was found in our study, which is consistent with Wang's report [38]. The controversies on the relationship of IOP and refraction condition need to be further studied in the future.

### Correlation between CCT and IOP values

Numerous studies have elaborated the positive relation of CCT and measured IOP, and the quantitative relation between them in both adult and adolescents population has also been revealed in many reports [6], [11], [14], [19], [39], [40], [41].Our study showed that the measured IOP increases 0.32 mmHg for every 10 µm increase in CCT, which is the results of Heidary [11].

The accurate IOP measurement is vital for the early diagnosis and timely treatment of glaucoma. Nowadays the non-contact tonometer is commonly used in clinical practice. However, the results of non-contact tonometer would overestimate the IOP in cases with thick CCT which leads to a misdiagnosis of glaucoma, and underestimate the IOP in cases with thin CCT which leads to a miss-diagnosis of glaucoma. Considering the effect of CCT on the measured IOP, we utilized and evaluated the CCT compensated IOP measurement mode of NT-530P, by which we can more accurately predict the actual IOP through the NCT results. Previous studies had verified the accuracy and reproducibility of measurement by NT-530P, and revealed an agreement between the NT-530P and Goldmann [42], [43], [44], [45]. This instrument provides the corrected IOP which decrease the effect of corneal thickness on IOP. Another advantages of NT-530p is non-contact, no injury, and easily accepted by patients.

One of the limitations of present study is that we only examined children aged 7 to18 years, as the children younger than 7 years old have not been recruited due to their bad compliance. Another limitation is that we used a non-contact IOP measuring method considering the NT-530P is time-saving and leads to a good compliance of targeted subjects. Although the accuracy of NT-530P has been verified previously, the Goldmann tonometry is still an ideal measurement as it's the widely accepted global standard.

All in all, this study revealed the quantitative relation of age, CCT, and IOP in children population in a specific area of northern China, by which we can better predict the changes of IOP in children and understand the effect of racial background on the IOP condition in adolescences.
